# The Unique Contributions of Verbal Analogical Reasoning and Nonverbal Matrix Reasoning to Science and Maths Problem‐Solving in Adolescence

**DOI:** 10.1111/mbe.12212

**Published:** 2019-08-16

**Authors:** Annie Brookman‐Byrne, Denis Mareschal, Andrew K. Tolmie, Iroise Dumontheil

**Affiliations:** ^1^ Centre for Brain and Cognitive Development, Department of Psychological Sciences Birkbeck, University of London; ^2^ Centre for Educational Neuroscience University of London; ^3^ Department of Psychology and Human Development, UCL Institute of Education University College London

## Abstract

Relational reasoning, the ability to detect meaningful patterns, matures through adolescence. The unique contributions of verbal analogical and nonverbal matrix relational reasoning to science and maths are not well understood. Functional magnetic resonance imaging data were collected during science and maths problem‐solving, and participants (*N* = 36, 11–15 years) also completed relational reasoning and executive function tasks. Higher verbal analogical reasoning associated with higher accuracy and faster reaction times in science and maths, and higher activation in the left anterior temporal cortex during maths problem‐solving. Higher nonverbal matrix reasoning associated with higher science accuracy, higher science activation in regions across the brain, and lower maths activation in the right middle temporal gyrus. Science associations mostly remained significant when individual differences in executive functions and verbal IQ were taken into account, while maths associations typically did not. The findings indicate the potential importance of supporting relational reasoning in adolescent science and maths learning.

Relational reasoning is the ability to consider relations between multiple mental representations (Crone et al., 2009) and detect meaningful patterns (Alexander, Dumas, Grossnickle, List, & Firetto, 2016). Relational reasoning can occur in the verbal domain, for example, in verbal analogical reasoning tasks (e.g., Leech, Mareschal, & Cooper, 2007; Richland & Burchinal, 2013), or in the visuospatial domain, for example, in nonverbal matrix reasoning tasks (e.g., Alexander et al., 2016). Supporting relational reasoning in science and maths classrooms may help students to learn better in these disciplines (Richland, Zur, & Holyoak, 2007; Vendetti, Matlen, Richland, & Bunge, 2015). There is cross‐sectional and longitudinal evidence of associations between individual differences in relational reasoning and maths (e.g., Dumontheil & Klingberg, 2012; Green, Bunge, Briones Chiongbian, Barrow, & Ferrer, 2017). However less is known regarding the link between individual differences in relational reasoning and science, with studies thus far focusing on the importance of relational reasoning in the learning of scientific material (Dumas, Alexander, & Grossnickle, 2013). Relational reasoning and the underlying neural systems show prolonged development which continues into adolescence (Dumontheil, 2014), a time when pupils are presented with increasingly complex science and maths concepts and problems (Department for Education, 2013a, 2013b). Establishing the roles of different aspects of relational reasoning, in different domains, namely verbal analogical reasoning and nonverbal matrix reasoning, in science and maths problem‐solving during adolescence may lead to more concrete advice for secondary school educators.

The (left) rostrolateral prefrontal cortex (RLPFC; Brodmann Area [BA] 10/47) has been proposed to play a specific role in relational integration (Bunge, Helskog, & Wendelken, 2009), whether the information being manipulated is semantic or visuospatial (Wendelken, Chung, & Bunge, 2012). In addition, the processing of single relations and relational integration rely on a network including the dorsolateral prefrontal cortex (DLPFC; BA9/46), ventrolateral prefrontal cortex (VLPFC; BA 45), and the parietal cortex (BA 7/40) (Wendelken et al., 2012), across childhood, adolescence, and adulthood (Crone et al., 2009; Dumontheil, Houlton, Christoff, & Blakemore, 2010; Whitaker, Vendetti, Wendelken, & Bunge, 2017). Over the course of development there is evidence of increased specialization of the RLPFC for relational integration, versus the manipulation of single relations (e.g., Crone et al., 2009; Dumontheil et al., 2010; Wendelken, O'Hare, Whitaker, Ferrer, & Bunge, 2011; see Dumontheil, 2014 for review). In addition, developmental changes in RLPFC structure and functional connectivity associate with changes in relational reasoning performance during adolescence (Bazargani, Hillebrandt, Christoff, & Dumontheil, 2014; Dumontheil et al., 2010; Wendelken, Ferrer, Whitaker, & Bunge, 2016).

A number of cross‐sectional studies have provided evidence for a link between nonverbal matrix reasoning and maths performance, such as in 5‐ to 19‐year olds (Taub, Floyd, Keith, & McGrew, 2008) and 15‐ to 16‐year olds (Kyttälä & Lehto, 2008). Stronger evidence comes from longitudinal studies. One study found that nonverbal matrix reasoning was a predictor of maths performance in 6‐ to 16‐year olds 2 years later (Dumontheil & Klingberg, 2012). Another found that in 6‐ to 21‐year olds fluid reasoning (including nonverbal tests of matrix reasoning, analysis synthesis, and concept formation) was a greater predictor of maths 18 months later than previous maths performance (Green et al., 2017). A third study revealed that a combined measure of relational reasoning, which included numerical reasoning, verbal analogical reasoning, and spatial reasoning, predicted maths learning in 11‐ to 14‐year olds over 2 years (Primi, Ferrão, & Almeida, 2010). Other studies have shown teaching by analogy to improve maths performance in adults (Richland & McDonough, 2010), and science performance in 9‐ to 10‐year olds (Matlen, Vosniadou, Jee, & Ptouchkina, 2009) and adults (Jee et al., 2013).

These findings have led to suggestions that relational reasoning may play a key role for maths development (Green et al., 2017; Miller Singley & Bunge, 2014). First, relational reasoning skills are likely to play a role in science and maths reasoning during problem‐solving, by allowing individuals to extract the relations between key elements of a given problem, compare and integrate them into a solution. Whether verbal‐semantic or visuospatial relational reasoning skills are recruited likely depends on the way the problem is presented. Second, relational reasoning skills have been proposed to support maths conceptual learning, by allowing a gradual build‐up of understanding of relations between, for example, single digit numbers, fractions, and equations with abstract terms (Miller Singley & Bunge, 2014), as well as by allowing understanding of concepts through analogies (Vendetti et al., 2015). Emphasizing and scaffolding the use of relational reasoning in the classroom therefore may lead to improved conceptual knowledge acquisition and problem‐solving (Miller Singley & Bunge, 2014; Vendetti et al., 2015).

Science and maths problem‐solving are supported by domain‐specific factual knowledge, procedural skills, and conceptual understanding (e.g. Cragg & Gilmore, 2014; Zimmerman, 2000). Although little research has focused on specific associations with these three components (although see Cragg, Keeble, Richardson, Roome, & Gilmore, 2017), a range of cognitive functions have been shown to associate with individual differences in science and maths achievement more broadly in late childhood or adolescence: these include spatial ability (Hodgkiss, Gilligan, Tolmie, Thomas, & Farran, 2018), vocabulary (Donati, Meaburn, & Dumontheil, 2019), processing speed (Donati et al., 2019), and executive functions (Cragg et al., 2017; Cragg & Gilmore, 2014), including inhibitory control (Brookman‐Byrne, Mareschal, Tolmie, & Dumontheil, 2018; Gilmore, Keeble, Richardson, & Cragg, 2015; Khng & Lee, 2009) and working memory (Donati et al., 2019; Kyttälä & Lehto, 2008; Rhodes et al., 2014; Rhodes et al., 2016). Since relational reasoning also associates with executive functions (Richland & Burchinal, 2013), any links between relational reasoning and science and maths may be driven by shared reliance on executive functions. Controlling for executive function is therefore important in considering the link between relational reasoning and science and maths performance.

In the current study, we first investigated behavioral associations between verbal analogical reasoning and nonverbal matrix reasoning and science and maths problem‐solving while controlling for possible shared associations with executive functions. Second, we examined whether individual differences in relational reasoning associated with individual differences in brain activation during science and maths problem‐solving, since neural data can reveal associations not seen in behavioral data alone (Dumontheil, Wolf, & Blakemore, 2016).

Secondary school participants aged 11–15 years solved science and maths problems while functional magnetic resonance imaging (fMRI) data were collected. Participants also completed tests of verbal analogical reasoning, nonverbal matrix reasoning, response inhibition, semantic inhibition, visuospatial working memory (VSWM), and verbal working memory (VWM). We predicted that better relational reasoning on both tasks would be associated with better science and maths performance (higher accuracy and faster reaction times (RTs)), when controlling for executive functions. We predicted that higher relational reasoning scores on both tasks would be associated during science and maths problem‐solving with greater recruitment of brain regions involved in relational reasoning, namely the RLPFC (BA 10/46), DLPFC (BA 9/46), VLPFC (BA 45/47), and parietal cortex (BA 7/40).

In terms of type of relational reasoning, we predicted that verbal analogical reasoning would be more important in science, since the language requirements are greater in science than maths; it is thought that verbal encoding of associations is a key skill in science learning (Tolmie, Ghazali, & Morris, 2016). Reversely, we predicted that nonverbal matrix reasoning would be more important in maths, which requires less language and more visuospatial processing, in line with the previous research, albeit with a younger sample (van der Sluis, de Jong, & van der Leij, 2007).

## METHODS

### Participants

Thirty‐eight participants (20 girls and 18 boys) aged 11–15 years, with no known neurological or developmental disorders, from schools in a range of demographic areas, took part. Written informed parental and participant consent was obtained in accordance with the guidelines of the local ethics committee, which approved the study. Participants were given pictures of their brain and £20, and travel expenses were reimbursed. One participant was excluded due to low accuracy in the science and maths task (15‐year old girl), and another because of movement in the science and maths task (12‐year old girl). The final sample consisted of 18 girls and 18 boys (age range = 137–185 months, *M* = 161, *SD* = 16).

### Tasks

#### *Science and Maths*

**Figure 1:**
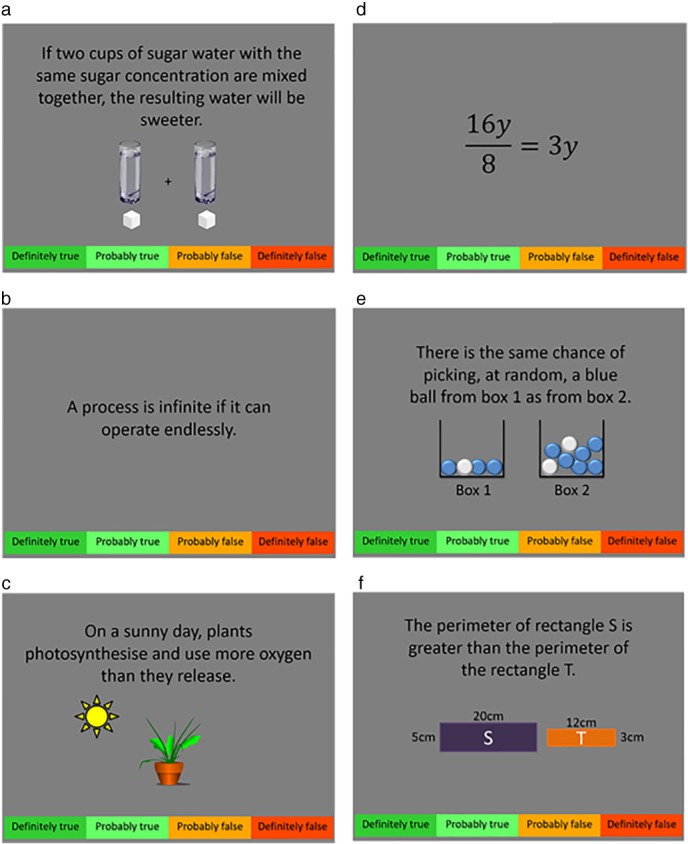
Example science **(**a–c**)** and maths **(**d–f**)** problems. Participants judged whether each statement was definitely true, probably true, probably false, or definitely false, by pressing one of four buttons with their index and middle fingers. A time limit of 12 s was imposed, with a warning appearing at 9 s to encourage participants to answer. Each participant answered 96 problems, of which half were science and half were maths. Half of the problems in each discipline were true and half were false. All problems were relevant to Key Stage 3 for England curricula in science and maths. Problems varied in difficulty, with half of the problems targeting a common counterintuitive concept (a, c, e). Note that text size has been increased here to enhance legibility. All stimuli and a detailed description of the task are available online: https://osf.io/ytcwk/.

The science and maths task was adapted from Brookman‐Byrne et al. (2018). Participants were shown science and maths statements relating to a wide range of topics from school curricula in England, and judged whether they were true or false by pressing one of four buttons (Figure 1 for more information).

Stimuli appeared in four alternating runs of separate science and maths trials, with the first topic counterbalanced across participants. Each run comprised different stimuli and included 24 trials, each lasting 16 s. Stimuli remained on screen until a response was given or 12 s had passed, at which point participants were presented either with a fixation cross (1/3 of trials) or an active baseline task (2/3 of trials) to keep participants engaged during delays between trials, while moving their attention away from the problems. Participants saw one of two sets of 96 problems. Cronbach's alpha and Spearman‐Brown split‐half reliability were calculated in SPSS for each set, demonstrating acceptable reliability given that the items were intentionally disparate and included both disciplines (Cronbach's alpha = .66 and .83, Spearman‐Brown coefficient = .87 and .76). The active baseline presented a series of arrows pointing left or right, and participants pressed the corresponding key. A central fixation cross appeared for 10 s at the start and end of each run, and 15 s in the middle of each run. The task lasted approximately 30 min in total. Accuracy and RT were recorded. Stimuli and a detailed task description are available online (https://osf.io/4saeu/).

#### *Relational Reasoning*

A verbal analogical reasoning task adapted from Leech et al. (2007) was administered on a laptop using a Google Form. Twenty‐four questions were presented, with four response options to choose from (e.g., Nose is to Smelling as Eye is to… Stink/Glasses/Seeing/Listening). The number of correct responses was recorded.

Raw scores from the Matrix Reasoning subtest of the WASI‐II (Wechsler, 2011) provided a measure of nonverbal matrix reasoning ability.

#### *Executive Functions*

Two inhibitory control tasks were administered (see Appendix S1, Supporting Information). The Go/No‐Go, adapted from Watanabe et al. (2002), measured simple and complex response inhibition. Key measures were RT costs in Go trials of the presence of No‐Go trials in the simple and complex blocks. The numerical Stroop, adapted from Khng and Lee (2014), measured semantic inhibition. Key measures were accuracy and RT costs in incongruent compared to congruent trials.

The Dot Matrix test of the Automated Working Memory Assessment was adapted from Alloway (2007), measuring VSWM. VWM was assessed using a backwards digit span. The total number of correct trials was recorded for each task.

#### *Verbal IQ*

Raw scores from the Vocabulary subtest of the WASI‐II (Wechsler, 2011) provided a measure of verbal IQ.

#### *Procedure*

Practices of the fMRI tasks were given first. The fMRI procedure lasted approximately 50 min; participants first completed the science and maths task, then a structural scan, then the Go/No‐Go and finally the numerical Stroop. Behavioral tasks took approximately 30 min in total, and were administered in a quiet room before or after scanning.

## STATISTICAL ANALYSIS

### Behavioral Analysis

Repeated measures ANOVAs were run on science and maths accuracy and RT. Analyses relating to the other tasks are reported in Appendix S1. Correlations were run between key variables. Hierarchical multiple regressions investigated the extent to which relational reasoning could account for individual differences in science and maths accuracy and RT by discipline. Block 1 variables included the control variables, inserted stepwise so that only significant predictors were kept in the model: verbal IQ, VSWM, VWM, simple Go RT cost, complex Go RT cost, Stroop accuracy cost, Stroop RT cost. Block 2 contained the relational reasoning measures entered stepwise: verbal analogical reasoning, nonverbal matrix reasoning.

### MRI Analysis

Detailed descriptions of MRI acquisition and preprocessing are reported in Appendix S1. Scanning runs were treated as separate time series, each of which was modeled by a set of regressors in the general linear model (GLM). Science and maths trials in each run were modeled by box‐car regressors using each trial's RT as the duration, and arrows blocks were modeled using 16 s minus each preceding trial's RT as the duration. All regressors were convolved with a canonical haemodynamic response function and, together with the separate regressors representing each censored volume and the session mean, comprised the full model for each run. Coordinates are given in Montreal Neurological Institute (MNI) space, region labelling was completed with Automated Anatomical Labelling (Tzourio‐Mazoyer et al., 2002), and BA labelling with MRIcron (Rorden & Brett, 2000).

First‐level contrasts of science and maths trials versus the arrows task (Science > Arrows; Maths > Arrows) were calculated. Contrasts were entered into one sample *t*‐tests to create SPM maps thresholded at *p* < .001 uncorrected at the voxel level and at family‐wise error (FWE) corrected *p* < .05 at the cluster level. Peak voxels significant at FWE corrected *p* < .05 at the voxel level are also indicated. Associations between blood‐oxygen‐level dependent (BOLD) signal and relational reasoning performance were investigated by running separate whole‐brain regressions entering either verbal analogical reasoning or nonverbal matrix reasoning as a regressor.

Follow‐up analyses (see Appendix S1) assessed whether associations remained after controlling for significant behavioral factors. Additionally, whole‐brain multiple regressions were performed.

## RESULTS

### Behavioral Results

ANOVAs showed that accuracy was higher and RTs faster in science (accuracy = 75.0% [*SD* = 1.5], RT = 5,675 ms [*SD* = 139]) than maths (accuracy = 71.0% [*SD* = 1.4], RT = 6,191 ms [*SD* = 141]), *p'*s < .004. Participants completed an average of 468 arrows trials, with a mean accuracy of 84.4% (*SD* = 0.2), and mean RT for correct trials of 501 ms (*SD* = 41).

Correlations (Table 1) showed that those with better verbal analogical reasoning were more accurate and faster in both disciplines. Those with better nonverbal matrix reasoning were more accurate in science. Verbal IQ correlated with science and maths, and verbal analogical reasoning. VSWM correlated with both relational reasoning measures but not science or maths, while VWM correlated with nonverbal matrix reasoning and science accuracy. Higher simple Go RT cost associated with verbal analogical reasoning and science accuracy.

**Table 1:** Pearson Correlation Coefficients of Regression Variables for Science and Maths by Discipline

Variable	1	2	3	4	5	6	7	8	9	10	11	12	13
1. Verbal analogical reasoning													
2. Nonverbal matrix reasoning	**.52** **												
3. Science accuracy	**.69** ***	**.35** *											
4. Science RT	**−.49** **	−.28	−.21										
5. Maths accuracy	**.50** **	.23	**.62** ***	−.31									
6. Maths RT	**−.39** *	−.12	−.13	**.73** **	−.29								
7. Simple Go RT cost	**.35** *	.21	**.38** *	−.30	.01	−.12							
8. Complex Go RT cost	.12	.01	.07	−.32	.04	−.09	**.57** ***						
9. Stroop accuracy cost	.18	.10	.10	.03	.20	.01	−.03	−.04					
10. Stroop RT cost	−.11	.19	−.16	−.05	.05	.14	.01	.13	−.16				
11. VSWM^a^	**.36** *	**.44** **	.30	−.23	.33	−.25	.09	−.01	−.01	.21			
12. VWM	.24	**.45** **	**.40** *	−.26	.24	−.19	.23	−.05	−.07	.19	**.44** **		
13. Verbal IQ	**.51** **	.20	**.64** ***	−.**36** *	**.53** **	−.22	.30	.25	.15	−.23	.06	.14	
14. Age (months)	−.01	−.14	.33	.03	**.37** *	−.19	.03	.06	.13	−.06	.15	−.10	**.37** *

*Note*. Statistically significant (two‐tailed) correlations are highlighted in bold. RT = reaction time; VSWM = visuospatial working memory; VWM = verbal working memory.

a Reduced sample size of 35 participants.

* *p* < .05

** *p* < .01

*** *p* < .001.

The first regression investigated whether relational reasoning could account for individual differences in science accuracy when relevant verbal IQ and executive function differences were taken into account. Model 1a selected verbal IQ (*R* ^2^ = 41.7%, *p* < .001), model 1b added VWM (*ΔR* ^*2*^ = 10.9%, *p* = .011), and model 1c added verbal analogical reasoning (*ΔR* ^*2*^ = 11.9%, *p* = .003, Table 2). In maths, model 2a selected verbal IQ (*R* ^2^ = 28.2%, *p* = .001), model 2b added VSWM (*ΔR* ^*2*^ = 8.7%, *p* = .043), and no relational reasoning measures were selected (Table 2).

**Table 2:** Regression Models for Science and Maths Accuracy

Dependent variables	Independent variables	*β*	*t*	*p*
Science accuracy				
Model 1a: *F*(1, 34) = 23.63, *p* < .001, *R* ^2^ = 41.7%	Constant		0.39	.701
	**Verbal IQ**	**.65**	**4.86**	**< .001**
Model 1b: *F*(2, 34) = 17.75, *p* < .001, *R* ^2^ = 52.6%	Constant		0.24	.180
	**Verbal IQ**	**.60**	**4.89**	**< .001**
	**VWM**	**.33**	**2.71**	**.011**
Model 1c: *F*(3, 34) = 18.76, *p* < .001, *R* ^2^ = 64.5%	Constant		0.02	.987
	**Verbal IQ**	**.40**	**3.15**	**.004**
	**VWM**	**.25**	**2.26**	**.031**
	**Verbal analogical reasoning**	**.42**	**3.22**	**.003**
Maths accuracy				
Model 2a: *F*(1, 34) = 12.97, *p* = .001, *R* ^2^ = 28.2%	Constant		1.00	.325
	**Verbal IQ**	**.53**	**3.60**	**.001**
Model 2b: *F*(2, 34) = 9.37, *p* = .001, *R* ^2^ = 36.9%	Constant		0.76	.451
	**Verbal IQ**	**.51**	**3.64**	**.001**
	**VSWM**	**.30**	**2.10**	**.043**
Science RT				
Model 3a: *F*(1, 34) = 4.74, *p* = .037, *R* ^2^ = 12.6%,	**Constant**		**5.62**	**< .001**
	**Verbal IQ**	**−.36**	**−2.18**	**.037**
Model 3b: *F*(2, 34) = 5.64, *p* = .008, *R* ^2^ = 26.0%,	**Constant**		**6.22**	**< .001**
	Verbal IQ	−.13	−0.73	.470
	**Verbal analogical reasoning**	**−.43**	**−2.42**	**.022**
Maths RT				
Model 4a: *F*(1, 34) = 5.41, *p* = .026, *R* ^2^ = 14.1%	**Constant**		**8.58**	**< .001**
	**Verbal analogical reasoning**	**−.38**	**−2.33**	**.026**

*Note*. Significant predictors (*p* < .05) are highlighted in bold. β = standardized coefficients; RT = reaction time; VSWM = visuospatial working memory; VWM = verbal working memory.

Regressions performed on science RT showed that model 3a selected verbal IQ (*R* ^2^ = 12.6%, *p* = .037), model 3b added verbal analogical reasoning (*ΔR* ^*2*^ = 13.5%, *p* = .022), with verbal IQ no longer significant. In maths, verbal analogical reasoning was the only significant predictor (Table 2).

Although the sample is small, age correlated with maths accuracy and verbal IQ (*r*'s = .37). Regressions were repeated controlling for age; this did not change the pattern of results.

### FMRI Results

**Figure 2:**
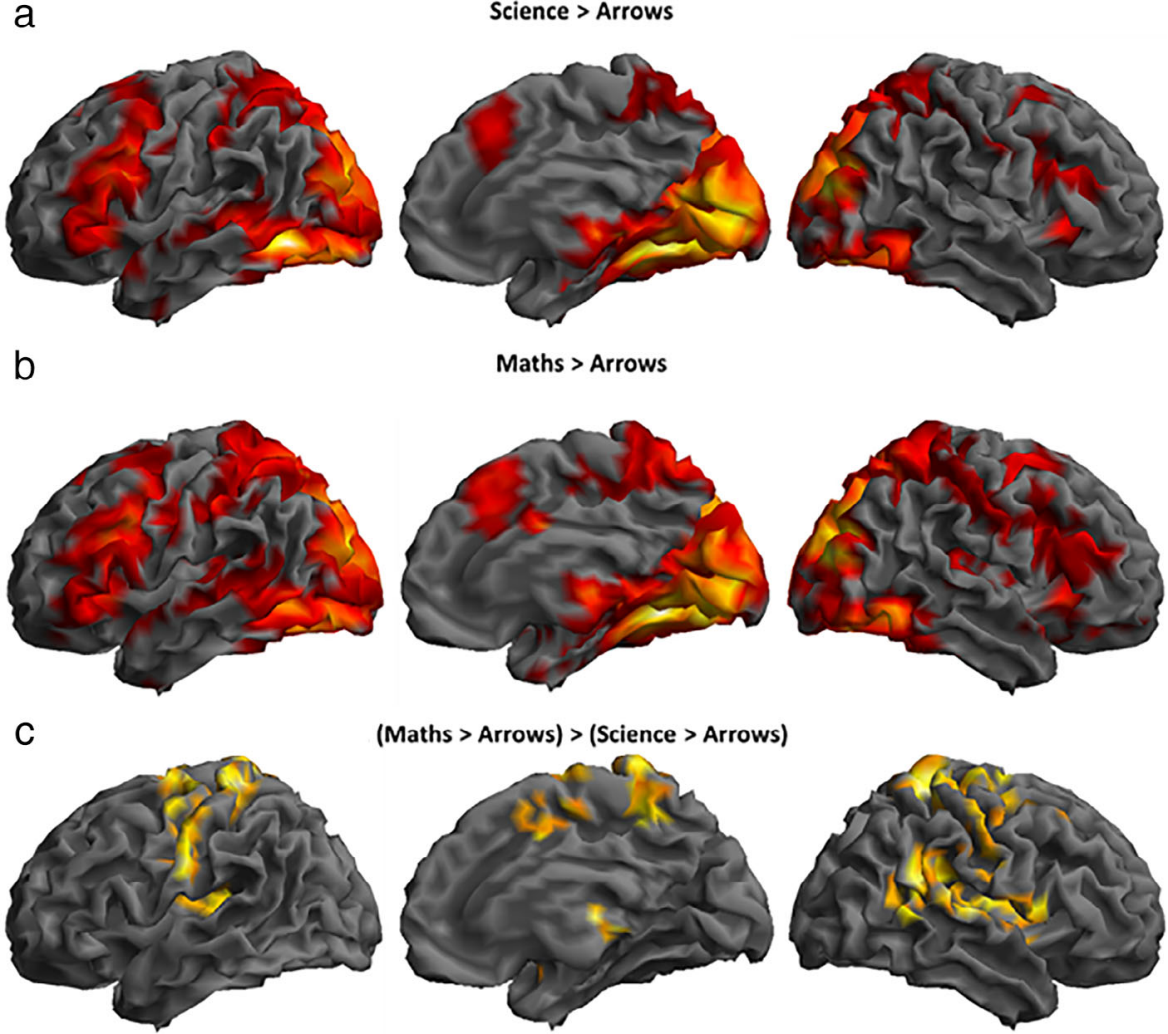
Regions of increased BOLD signal in the **(**a**)** Science > Arrows contrast, **(**b**)** Maths > Arrows contrast, and **(**c**)** (Maths > Arrows) > (Science > Arrows) contrast from the one sample *t*‐tests with no covariates added. In both disciplines, there was extensive bilateral activation covering most of the occipital cortex, superior and inferior parietal gyri and the precuneus, the pre‐supplementary motor area and posterior parts of the superior and middle frontal gyri, the anterior insulae, posterior parts of the inferior and middle temporal gyri, the posterior parts of the hippocampi and parahippocampal gyri, and finally subcortically parts of the thalamus and caudate. In addition, there was mostly left‐lateralized activation of the precentral and inferior frontal gyri, and activation in the left middle temporal gyrus extending into the anterior temporal cortex. Maths problems were associated with increased BOLD signal bilaterally in the pre‐ and postcentral gyri, the supplementary motor area and middle cingulate cortex, the thalamus, and, mostly in the right hemisphere inferior frontal gyrus, superior temporal gyrus and parts of the occipital cortex. *p*_uncorr_ < .001 at the voxel level, *p*_FWE_ < .05 at the cluster level. Images are rendered on the canonical brain in SPM, showing from left to right: the lateral view of the left hemisphere, and medial and lateral views of the right hemisphere. Contrasts are available online: https://osf.io/ytcwk/.

Both the Science > Arrows (Figure 2a) and the Maths > Arrows (Figure 2b) contrasts showed increased BOLD signal in a broad bilateral network of regions. There was greater BOLD signal in a range of regions in maths compared to science (Figure 2c). No regions showed greater activation for science than maths.

**Figure 3:**
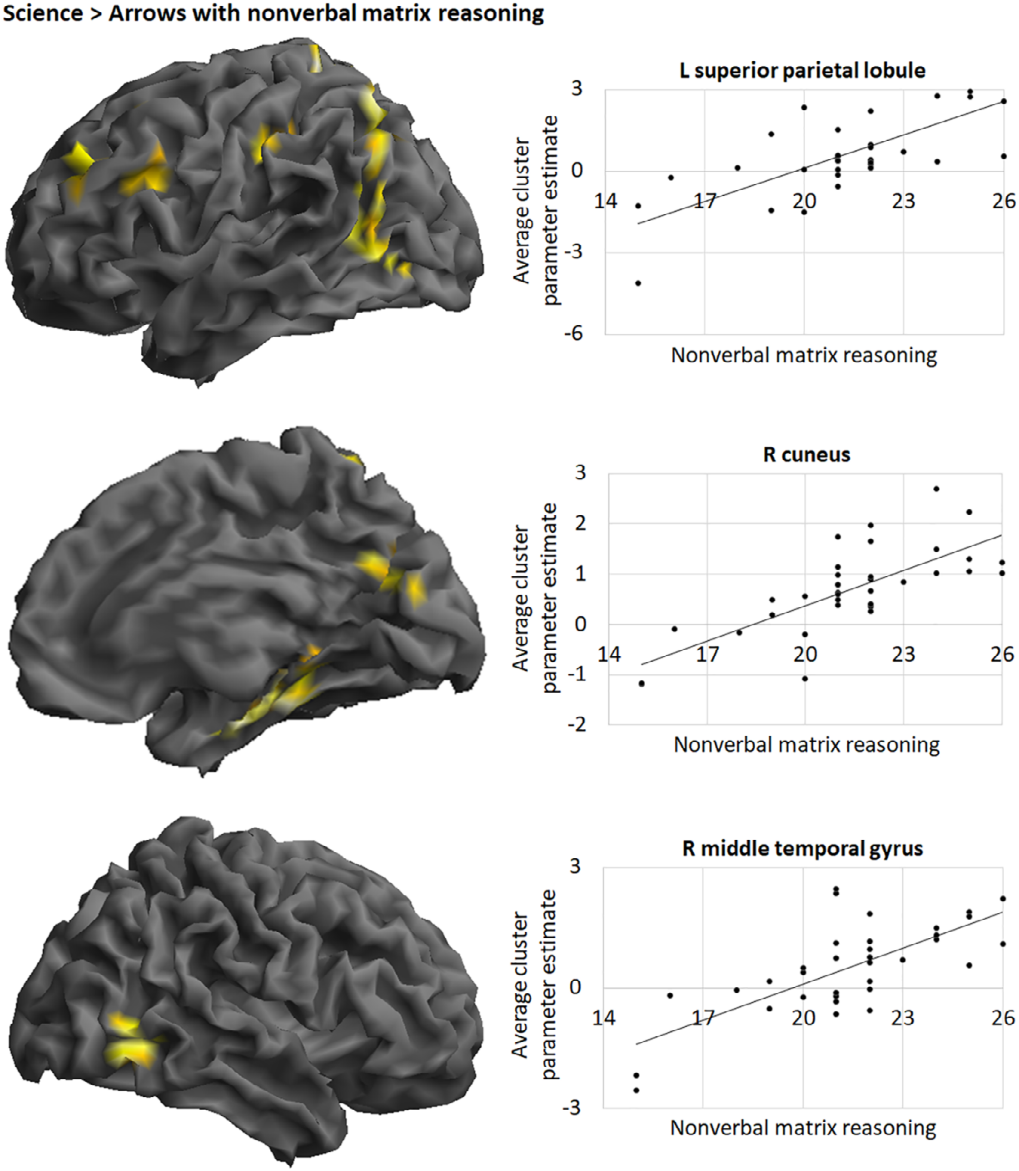
Brain regions where BOLD signal during science problem‐solving positively correlated with nonverbal matrix reasoning, showing from top to bottom: the lateral view of the left hemisphere, and medial and lateral views of the right hemisphere. Three clusters have been chosen to demonstrate the positive association between average parameter estimates and nonverbal matrix reasoning on illustrative scatterplots. Contrasts *p*_uncorr_ < .001 at the voxel level and *p*_FWE_ < .05 at the cluster level. Images are rendered on the canonical brain in SPM. L = left; R = right. R cuneus refers to the whole cluster including the L and R precuneus.

**Figure 4:**
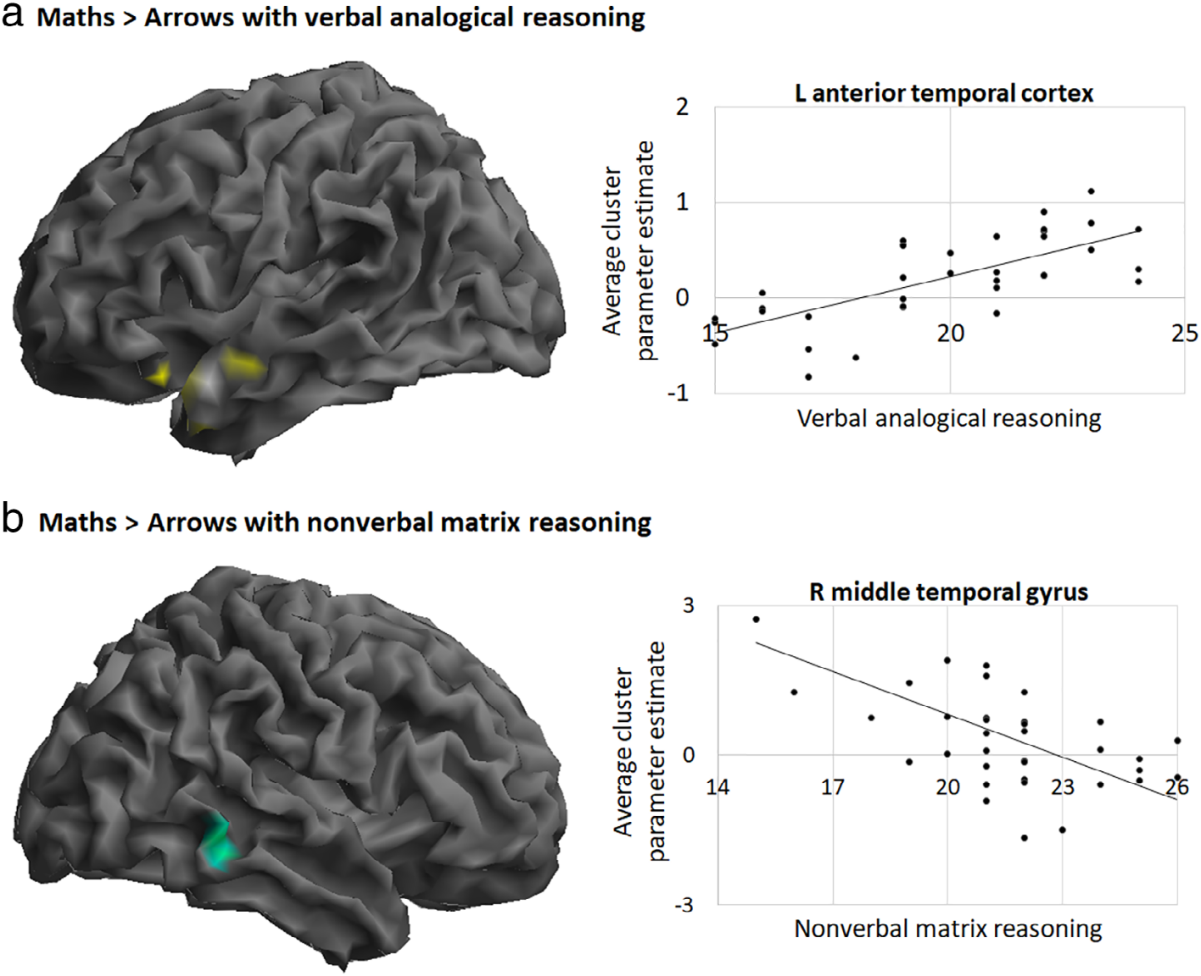
Brain regions where BOLD signal during maths problem‐solving **(**a**)** positively correlated with verbal analogical reasoning (shown in yellow) and **(**b**)** negatively correlated with nonverbal matrix reasoning (shown in green), with corresponding scatterplots. Contrasts *p*_uncorr_ < .001 at the voxel level and *p*_FWE_ < .05 at the cluster level. Images are rendered on the canonical brain in SPM.

Nonverbal matrix reasoning, but not verbal analogical reasoning, was a significant covariate of the Science > Arrows contrast, with higher nonverbal matrix reasoning associated with higher BOLD signal in parietal, frontal and temporal cortex clusters (Figure 3 **,** Table 3). Higher verbal analogical reasoning associated with higher BOLD in the left anterior temporal cortex in the Maths > Arrows contrast, while higher nonverbal matrix reasoning associated with lower BOLD in right middle temporal gyrus (Figure 4 **,** Table 4). Plotted average parameter estimates indicate that these associations were not due to outliers but general trends across participants.

**Table 3:** Regions Where BOLD Signal in the Science > Arrows Contrast Positively Correlated with Nonverbal Matrix Reasoning

			MNI				
Brain region	L/R	BA	*x*	*y*	*z*	*Z*‐score	Cluster size
Fusiform gyrus	L	30	−27	−28	−28	5.52*	1,141**
Lobule III of vermis	R	30	6	−46	−22	5.00*	
Cerebellum	L		−9	−55	−31	4.87*	
Superior parietal lobule	L	7	−30	−64	53	4.43	169**
Angular gyrus	L	39	−39	−64	35	4.32	
Precuneus	L	18	−6	−67	29	4.36	192**
Cuneus	R	18	21	−73	26	4.22	
Precuneus	R	23	9	−67	29	3.92	
Paracentral lobule	L	4	−3	−34	59	4.35	264**
Precuneus	L	5	−12	−46	68	4.34	
Midcingulate area	L	5	−3	−40	50	4.33	
Middle temporal gyrus	L	39	−36	−55	14	4.30	270**
Middle temporal gyrus	L	37	−45	−61	5	4.30	
Middle temporal gyrus	L	39	−54	−67	17	4.22	
Middle temporal gyrus	R	37	54	−58	−1	4.30	146**
Calcarine sulcus	R	19	33	−67	2	3.74	
Inferior parietal lobule	L	40	−33	−34	38	4.03	104**
Inferior parietal lobule	L	48	−42	−28	35	3.81	
Supramarginal gyrus	L	44	−51	−25	41	3.79	
Middle frontal gyrus	L	8	−27	11	53	3.89	169**
Inferior frontal gyrus	L	48	−33	23	29	3.75	
Inferior frontal gyrus	L	48	−42	20	26	3.54	
Superior frontal gyrus	L	9	−27	41	38	3.62	83**
Superior frontal gyrus	L	9	−21	38	32	3.57	
Middle frontal gyrus	L	46	−39	44	23	3.34	

*Note*. L = left; R = right; BA = Brodmann area; MNI = Montreal Neurological Institute.

* *p*_FWE_ < .05 at the voxel‐level.

** *p*_FWE_ < .05 at the cluster‐level (cluster defining threshold: *p*_uncorr_ < .001).

**Table 4:** Regions Where BOLD Signal in the Maths > Arrows Contrast Positively Correlated with Verbal Analogical Reasoning and Negatively Correlated with Nonverbal Matrix Reasoning

			MNI				
Brain region	L/R	BA	*x*	*y*	*z*	*Z*‐score	Cluster size
Maths > Arrows and verbal analogical reasoning							
Middle temporal gyrus	L	21	−51	−1	−19	4.70*	214**
Superior temporal pole	L	38	−42	14	−25	4.03	
Inferior temporal gyrus	L	20	−42	5	−34	3.99	
Maths > Arrows and nonverbal matrix reasoning							
Middle temporal gyrus	R	21	63	−40	−7	4.39	122**

*Note*. L = left, R = right, BA = Brodmann area, MNI = Montreal Neurological Institute.

* *p*_FWE_ < .05 at the voxel‐level.

** *p*_FWE_ < .05 at the cluster‐level (cluster defining threshold: *p*_uncorr_ < .001).

Follow‐up analyses (see Appendix S1) showed that when controlling for relevant performance measures, relational reasoning predicted additional variance in BOLD signal within the clusters identified. At the whole‐brain level, nonverbal matrix reasoning remained a significant predictor of activation in the Science > Arrows contrast in a subset of regions (cerebellum, left superior parietal lobule, and left middle temporal gyrus) when controlling for other variables. Neither of the relational reasoning measures predicted activation in the Maths > Arrows contrast when controlling for other variables.

## DISCUSSION

This study investigated the unique contributions of verbal analogical reasoning and nonverbal matrix reasoning to science and maths problem‐solving in adolescence. Verbal analogical reasoning was associated with higher accuracy and faster RTs in both science and maths, although the association with maths accuracy disappeared when verbal IQ and VSWM were taken account of. Nonverbal matrix reasoning was associated only with science accuracy, and this effect was not maintained after controlling for verbal IQ and VWM. Nonetheless, nonverbal matrix reasoning was related to broad activation during science problem‐solving, with three clusters remaining significant after controlling for other variables. Verbal analogical reasoning was positively associated with activation in the right anterior temporal cortex during maths problem‐solving, while nonverbal matrix reasoning was negatively associated to activation in the left middle temporal gyrus. Neither of these maths associations remained when controlling for other variables.

We predicted that both types of relational reasoning would be associated with higher accuracy and faster RTs in science and maths, and as such, our findings did not always meet our predictions. Further, we predicted that verbal analogical reasoning would be more important in science than maths, and this was supported by the behavioral analyses: correlations with science were higher, and the correlation with maths disappeared when verbal IQ and VSWM were controlled for. These results are in line with the suggestion that science learning requires verbal encoding of associations (Tolmie et al., 2016) and is supported by analogical reasoning (Jee et al., 2013; Matlen et al., 2009; Vendetti et al., 2015). Although participants recruited the RLPFC, DLPFC, VLPFC, and parietal cortex regions previously implicated in relational reasoning when resolving science problems, we did not observe the predicted correlation between activation in these regions and individual differences in verbal analogical reasoning scores. Our results further suggest that previous evidence linking analogical reasoning to maths (Alexander et al., 2016; White, Alexander, & Daugherty, 1998) may be in part attributable to executive functions and verbal IQ, since we saw this link disappear when individual differences in executive functions and verbal IQ were taken account of. The results highlight the importance of controlling for verbal IQ and executive functions when investigating associations with relational reasoning.

We hypothesized that nonverbal matrix reasoning would be more important in maths. This was not supported by the behavioral analyses, which showed that nonverbal matrix reasoning was only significantly related to science accuracy. This is in contrast to previous evidence that showed matrix reasoning measures to relate with maths (Dumontheil & Klingberg, 2012; Green et al., 2017; Kyttälä & Lehto, 2008; Wei, Yuan, Chen, & Zhou, 2012). The greater link between maths and verbal analogical reasoning compared to nonverbal reasoning differs from other research (van der Sluis et al., 2007). This may be due to the relatively high language requirements of the current maths problems, since the problems used by van der Sluis et al. (2007) were all arithmetic, requiring addition, multiplication, and subtraction. Nonetheless, the maths tasks in the previous literature that show a link with relational reasoning are varied, with some more verbal (Kyttälä & Lehto, 2008) and others less verbal (Dumontheil & Klingberg, 2012; Wei et al., 2012) in nature. It is also possible that the mismatch between the current results and the previous literature is due to the different ages of participants, with much of the previous literature pertaining to younger or older participants, or a very wide age range, or to a lack of sensitivity of the WASI Matrix Reasoning to individual differences.

Although nonverbal matrix reasoning was not associated with science behaviorally when controlling for other factors, it was positively associated with increased BOLD signal during science problem‐solving across a broad network. It is possible that those who were better at nonverbal matrix reasoning engaged those brain networks more during science problem‐solving, but they did not necessarily hold the knowledge necessary to get the answers correct. It is worth noting that other studies have shown behavioral and neuroimaging data may not map directly onto each other. This may be due to the sensitivity of different methods, since there are likely factors that influence behavioral data which might not be reflected in imaging data (Dumontheil et al., 2016). Of the hypothesized regions, only activation during science problem‐solving in the superior parietal lobule (BA 7) was associated with relational reasoning. Beyond its role in the manipulation of single relations and integration of relations (Crone et al., 2009; Dumontheil, 2014; Ferrer, O'Hare, & Bunge, 2009), this region is thought to be critical for the manipulation of information in working memory (Koenigs, Barbey, Postle, & Grafman, 2009). Importantly, the association remained when executive functions were controlled for, suggesting that it was not solely the requirement of working memory that led to individual differences in SPL activation.

During maths problem‐solving, increased BOLD signal in the left anterior temporal cortex was associated with better verbal analogical reasoning. This region is thought to be critical for semantic processing of conceptual knowledge (Pobric, Lambon Ralph, & Jefferies, 2009; Rice, Lambon Ralph, & Hoffman, 2015) and the construction of complex meaning (Westerlund & Pylkkänen, 2017). Recruitment of anterior temporal cortex may therefore reflect shared requirements for construction and processing of complex concepts during maths problem‐solving and verbal analogical reasoning. There was a negative association between activation in the right middle temporal gyrus and nonverbal matrix reasoning, such that those who performed better in nonverbal matrix reasoning recruited this region less during maths problem‐solving. There is some evidence that this posterior region of the middle temporal gyrus supports language and reading processing (Saur et al., 2008; Xu et al., 2015), so one possible interpretation is that participants who were better at nonverbal relational reasoning relied less on language processing to solve the maths problems. However, follow‐up whole brain analyses showed that these associations did not hold when covarying for the other measures. This indicates that the neural activations described here may reflect executive processes or verbal IQ.

It is possible that the neural activations reported for these contrasts reflect increased task difficulty. The multiple‐demand (MD) network is a system that refers to common recruitment of certain brain areas in response to cognitive challenge (Duncan, 2010). The system extends over regions of the prefrontal and parietal cortex, and incorporates the intraparietal sulcus, inferior frontal sulcus, anterior insula and frontal operculum, rostral prefrontal cortex, pre‐supplementary motor area, and anterior cingulate cortex (Duncan, 2010). There is no overlap between MD regions and those that showed associations with relational reasoning during maths problem‐solving in the present study, while in science, some regions that correlated with nonverbal matrix reasoning align with typical MD regions (superior parietal lobule (BA 7) and middle frontal gyrus (BA 8)). Overall, this suggests that activation in these regions may reflect cognitive demand common to science and nonverbal matrix reasoning.

A strength of this study was in using a broad range of science and maths problems relating to the school curriculum, ensuring that conclusions are related to classroom reasoning. It also considered relational reasoning over and above executive functions and verbal IQ to uncover unique contributions. Further establishing the nature of the association between different types of relational reasoning and science and maths problem‐solving may lead to recommendations for teaching and learning. If those with better relational reasoning also perform better in science and maths, this suggests that encouraging relational reasoning during science and maths problem‐solving may support the development of both skills concurrently. Since maths requires understanding difficult abstract concepts, teaching by analogy may support learning (Richland et al., 2007). This teaching approach would be similar to that already tested in studies of science learning (Jee et al., 2013; Matlen et al., 2009). Vendetti et al. (2015) emphasized the importance of supporting relational reasoning within science, arguing that explicit explanation of comparisons is essential, as teachers may assume that analogous relations are obvious, when they are not to learners. These suggested approaches highlight the importance of supporting a cognitive skill within the discipline, which is in contrast to largely unsuccessful attempts to improve discipline‐performance through training cognitive skills in isolation (Melby‐Lervåg & Hulme, 2013).

This study investigated different types of relational reasoning in science and maths problem‐solving within behavioral and neuroimaging data. Overall, verbal analogical reasoning predicted unique variance in science performance, with more limited behavioral, but some neural associations, in maths. Nonverbal matrix reasoning showed minimal behavioral associations, but was related to neural activation in science and maths. Associations between relational reasoning and science problem‐solving mostly remained after controlling for executive functions, while associations with maths problem‐solving typically disappeared, suggesting a unique role of relational reasoning in both science and maths.

## Supporting information


**Appendix S1**. Supporting information
