# Supplementary material for: The Unique Contributions of Verbal Analogical Reasoning and Nonverbal Matrix Reasoning to Science and Maths Problem‐Solving in Adolescence
Source: Mind Brain Educ. 2019 Aug 16;13(3):211–23. doi: 10.1111/mbe.12212 (PMC7189624; doi:10.1111/mbe.12212)
Supplement: Supplementary file 1 — Appendix S1. Supporting information [file MBE-13-211-s001.docx]

**The unique contributions of verbal analogical reasoning and nonverbal matrix reasoning to science and maths problem-solving in adolescence: Supporting information**

Here we present additional information relating to the methods and results.

**Methods**

**Inhibitory control tasks**

The inhibitory control tasks were carried out inside the magnetic resonance imaging (MRI) scanner but only behavioral data are reported here. Practices were completed prior to scanning to ensure the tasks were understood.

The Go/No-Go, adapted from Watanabe and colleagues (2002), measured response inhibition. The task had a block design, with blocks of Go trials (press on the side where the beige square appears), blocks of simple Go/No-Go trials (press on the side where the beige square appears, do not press for blue squares), and blocks of complex Go/No-Go trials (press on the side of the square when the colour matches the previous square’s, otherwise do not press). Go/No-Go blocks contained 50% Go trials and 50% No-Go trials. Practice blocks of 10 trials were repeated if more than one No-Go error was made in the Simple and Complex blocks, and if more than one Go error was made in Go blocks. Stimuli remained on the screen for 400 ms, followed by a fixation cross in the centre of the screen, which remained on the screen between 600 and 800 ms. A fixation cross appeared in the centre of the screen for 10 s at the start and end of each run, and 15 s in the middle of each run. The task was performed in a single run, with four repeats of each block type. Each block contained 20 trials, with location, interstimulus interval, and trial type pseudo-randomised per block. Each block lasted 22 s and the task lasted approximately 6 min in total. Go RT costs corresponding to the need to slow down to accurately inhibit responses in No-Go trials were calculated for the simple and complex versions by subtracting mean Go RT in Go blocks from mean Go RT in simple Go-No blocks and from mean Go RT in complex Go/No-Go blocks for correct trials. RT costs were used for further analysis because accuracy across trials was high, and it was anticipated that RT costs would be more sensitive to individual differences.

The numerical Stroop, adapted from Khng and Lee (2014), measured semantic inhibition. Participants indicated how many numbers (from 1 to 4) were presented on the screen. The task had a block design with blocks of congruent trials (e.g. “3 3 3”, “1”), and mixed blocks comprising half incongruent (e.g. “2 2 2”, “4”) and half congruent trials. Stimuli remained on the screen until a response was made or a maximum of 1.1 s had passed. Each trial lasted 1.5 s and the remainder of the trial was filled with a fixation cross in the centre of the screen. Blocks alternated and there were a total of five blocks of each type, with a fixed trial order across participants. Before the start of a block, participants were shown an instruction screen for 2 s indicating which block type they would be completing. There was 10 s of fixation at the beginning and end of a run, with 15 s fixation roughly in the middle of a run. The task lasted approximately 5 min. A familiarisation phase of eight trials of asterisks instead of digits was repeated if more than one error was made. A congruent practice of eight trials was repeated if more than one error was made. Finally, a practice mixed block of eight trials was repeated if more than two errors were made. Accuracy and RT costs were calculated by subtracting incongruent performance from congruent performance averaged from both block types for accuracy, and vice versa for RT in correct trials.

**Working memory tasks**

In the Dot Matrix task (measuring visuospatial working memory [VSWM]), participants observed dots appearing in turn on a grid, and clicked where the dots had appeared, in order. One participant had missing data on this task due to a technical problem. In the backwards digit span (measuring verbal working memory [VWM]), the experimenter read out a series of numbers and the participant verbally repeated the numbers in reverse order. Both working memory tasks started with a load of three items, increasing in sets of four trials until two incorrect answers were given at any one load. The total number of correct trials was recorded for each task.

**Statistical analysis**

Means, standard deviations, and ranges were calculated for VSWM, VWM, verbal analogical reasoning, and nonverbal matrix reasoning.

For the inhibitory control tasks mean reaction times (RTs) were calculated from correct trials only. Repeated measures ANOVAs investigated the difference between Trial types in the Go/No-Go task (Go in Go blocks, Go in Simple blocks, Go in Complex blocks, No-Go in Simple blocks, No-Go in Complex blocks) and in the numerical Stroop task (congruent in Congruent blocks, congruent in Mixed blocks, incongruent in Mixed blocks) for accuracy and RT (for Go trials only in Go/No-Go task). Main effects were followed up with Bonferroni-corrected pairwise comparisons.

**MRI acquisition, preprocessing, and follow-up analyses**

Brain imaging data were acquired on a 1.5 Telsa Siemens Avanto MRI scanner with a 30-channel head coil. Structural data were acquired with a T1-weighted magnetization-prepared rapid gradient-echo (MPRAGE) with 2x generalised autocalibrating partial parallel acquisition acceleration, lasting 5.5 min. Functional data were acquired in six runs using the Centre for Magnetic Resonance Research multiband echo-planar imaging sequence (Xu et al., 2013) 4x acceleration, leak block on (Cauley et al., 2014) repetition time (TR) = 1 s, echo time (TE) = 45 ms, comprising 44 slices covering most of the cerebrum, with a resolution of 3 x 3 x 3 mm^3^. The structural MPRAGE was typically acquired between the science and maths task and the inhibitory control tasks.

MRI data were preprocessed and analysed using SPM12 (www.fil.ion.ucl.ac.uk/ spm/software/spm12/). Functional images were realigned to the mean images after the first realignment in a two pass procedure using a second-degree B-spline interpolation to correct for movement during the session. The bias-field-corrected structural image was co-registered to the mean realigned functional image, and segmented on the basis of Montreal Neurological Institute (MNI) registered International Consortium for Brain Mapping tissue probability maps. Resulting spatial normalisation parameters were applied to the realigned images to obtain normalised functional images with a voxel size of 3 x 3 x 3 mm, which were smoothed with an 8 mm full-width at the half-maximum Gaussian kernel. Framewise displacement (FD) was calculated for each volume as a scalar measure of head motion across the six realignment estimates (Siegel et al., 2014). Volumes with an FD greater than 0.9 mm were censored and excluded from the general linear model (GLM) estimation by including a regressor of no interest for each censored volume. Scanning runs with more than 15% of volumes censored or a root mean square movement greater than 1.5 mm were excluded from the analysis. Two participants had one scanning run (out of four) excluded due to movement (12y girl, 15y boy).

**Regions of interest (ROI) analyses**

To better understand what may underlie observed associations, significant regions of activation were followed up by averaging the data for the Science > Arrows or Maths > Arrows contrast over each identified cluster using MarsBaR (Brett, Anton, Valabregue, & Poline, 2002). Multiple regression analyses in SPSS assessed whether associations remained after controlling for the effect of any significant factors, including science and maths performance. The following variables were entered stepwise in block 1: science or maths accuracy and RT, verbal IQ, VSWM, VWM, simple Go RT cost, complex Go RT cost, Stroop accuracy cost, Stroop RT cost. Block 2 contained the relevant relational reasoning measure stepwise.

**Whole-brain analyses**

Associations between blood-oxygen-level dependent (BOLD) signal and relational reasoning were followed up with whole-brain regressions that included all variables (science or maths accuracy and RT, verbal analogical reasoning, nonverbal matrix reasoning, verbal IQ, VSWM, VWM, simple Go RT cost, complex Go RT cost, Stroop accuracy cost, Stroop RT cost), to further establish the specificity of associations.

**Results**

**Behavioral results**

Descriptive statistics for VSWM, VWM, verbal analogical reasoning, and nonverbal matrix reasoning are reported in **Table S1**.

**Table S1.** Descriptive statistics in the visuospatial working memory (VSWM), verbal working memory (VWM), verbal analogical reasoning, and nonverbal matrix reasoning tasks.

| **Task** | ***M* (*SD*)** | **Range** |
| --- | --- | --- |
| VSWM | 8.5 (3.6) | 2-16 |
| VWM | 9.3 (3.9) | 2-18 |
| Verbal analogical reasoning | 19.8 (2.7) | 15-24 |
| Nonverbal matrix reasoning | 21.4 (2.7) | 15-26 |

The repeated measures ANOVA on accuracy in the Go/No-Go showed a significant effect of Trial type (**Table S2**). Pairwise comparisons showed significant differences (*p*’s < .009) between all trial types apart from between Go trials in Go blocks and Simple Go trials, and between Complex Go and Complex No-Go trials, *p*’s = 1. Accuracy was highest in Simple No-Go trials, followed by Go trials in Go blocks, then Simple Go trials, Complex Go trials, and finally Complex No-Go trials. For RT, there was a significant effect of Trial type (**Table S2**) with significant differences between all trial types, *p*’s < .001. RTs were fastest in Go blocks, then Simple Go trials, and slowest in Complex Go trials.

The repeated measures ANOVA on accuracy (**Table S2**) in the numerical Stroop showed a significant effect of Trial type, with significant differences between all trial types, *p*’s < .006. The highest accuracy was in Congruent trials in Mixed blocks, followed by Congruent trials in Congruent blocks, and finally Incongruent trials. For RT there was a significant effect of Trial type (**Table S2**) with significant differences between all trial types, *p*’s < .001. RTs were fastest in Congruent trials within Congruent blocks, then Congruent trials in Mixed blocks, then Incongruent trials.

**Table S2.** Accuracy and RT estimated marginal means in the inhibitory control tasks.

|  | **Accuracy (%)** | **RT (ms)** |
| --- | --- | --- |
|  | ***M (SE)*** | ***M (SE)*** |
| **Go/No-Go** | *F*(4, 140) = 24.39,  *p* < .001, η_p_^2^ = .411 | *F*(2, 70) = 150.16,  *p* < .001, η_p_^2^ = .811 |
| *Go trials* |  |  |
| Go blocks | 93.3 (0.8) | 430 (6) |
| Simple blocks | 92.8 (1.1) | 471 (6) |
| Complex blocks | 84.3 (1.5) | 509 (7) |
| *No-Go trials* |  |  |
| Simple blocks | 95.7 (0.8) | - |
| Complex blocks | 80.8 (2.4) | - |
| **Numerical Stroop** | *F*(2, 70) = 77.44,  *p* < .001, η_p_^2^ = .689 | *F*(2, 70) = 339.71,  *p* < .001, η_p_^2^ = .907 |
| *Congruent trials* |  |  |
| Congruent blocks | 89.7 (1.0) | 633 (10) |
| Mixed blocks | 93.5 (1.3) | 692 (10) |
| *Incongruent trials* |  |  |
| Mixed blocks | 75.9 (1. 8) | 786 (10) |

**Functional MRI results**

**ROI analyses**

Follow-up regression analyses of mean data in each cluster were performed in SPSS. Possible explanatory variables (science or maths accuracy and RT, verbal IQ, and executive functions) were entered stepwise in block 1, followed by the relational reasoning measure in block 2. The first models selected one or two variables from block 1 (VSWM in six models, VWM in four models, and simple Go RT cost in two models), and in all second models relational reasoning measures remained significant predictors of BOLD signal, *p*’s ≤ .004. Block 1 accounted for 16-34% of the variance, and the relational reasoning measure accounted for an additional 17-46% of the variance.

**Whole brain analyses**

Follow-up analyses investigated the specificity of associations observed between relational reasoning measures and brain activity during the science and maths task with whole-brain regressions including all cognitive variables as regressors. In the Science > Arrows contrast, nonverbal matrix reasoning remained a significant predictor of BOLD signal change in three clusters when controlling for the effects of the other variables: the cerebellum, the superior/inferior parietal lobule, and the middle temporal gyrus (**Table S3, Figure S1**).

In the Maths > Arrows contrast, neither verbal analogical reasoning nor nonverbal matrix reasoning remained significant predictors when controlling for maths performance, verbal IQ, executive functions, and the other relational reasoning measure.

These patterns of results were similar when only one relational reasoning measure was entered in the multiple regressions, and when age was entered as an additional regressor.

**Table S3.** Regions where BOLD signal in the Science > Arrows contrast positively correlated with nonverbal matrix reasoning while controlling for the effects of science accuracy, science RT, verbal analogical reasoning, verbal IQ, and executive functions, ^a^*p*_FWE_ < .05 at the voxel-level, ^b^*p*_FWE_ < .05 at the cluster-level (cluster defining threshold: *p*_uncorr_ < .001). L = left, R = right, BA = Brodmann area, MNI = Montreal Neurological Institute. Note that the sample includes the 35 participants with a full dataset.

| **Brain region** | **L/R** | **BA** | **MNI** | | | ***Z*-score** | **Cluster size** |
| --- | --- | --- | --- | --- | --- | --- | --- |
|  |  |  | ***x*** | ***y*** | ***z*** |  |  |
| Cerebellum | R |  | 12 | -73 | -28 | 5.36^a^ | 151^b^ |
| Cerebellum | R |  | 36 | -64 | -31 | 4.04 |  |
| Superior parietal lobule | L | 7 | -30 | -64 | 53 | 4.92^a^ | 109^b^ |
| Inferior parietal lobule | L | 40 | -48 | -46 | 50 | 3.62 |  |
| Middle temporal gyrus | L | 19 | -33 | -55 | 11 | 3.97 | 74^b^ |
| Middle temporal gyrus | L | 37 | -48 | -67 | 14 | 3.86 |  |


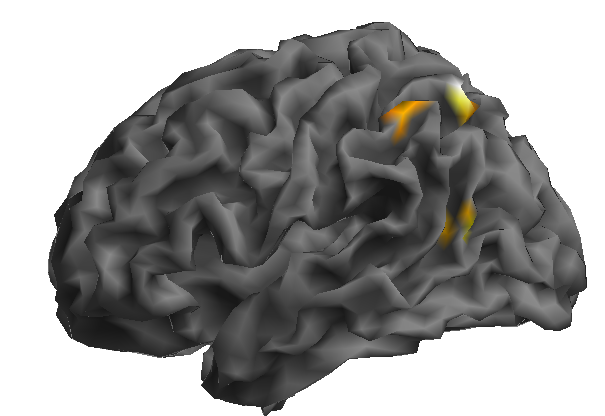


**Figure S1.** Regions where BOLD signal in the Science > Arrows contrast positively correlated with nonverbal matrix reasoning while controlling for the effects of science accuracy, science RT, verbal analogical reasoning, verbal IQ, and executive functions, ^a^*p*_FWE_ < .05 at the voxel-level, ^b^*p*_FWE_ < .05 at the cluster-level (cluster defining threshold: *p*_uncorr_ < .001)
